# Phenacetin in Rheumatism

**Published:** 1895-08

**Authors:** 


### SELECTIONS.

### Phenacetin in Rheumatism.

According to the *Journal de Medecine de Paris* for April 29, 1894, useful results are obtained in cases of acute rheumatism by applying phenacetin externally to the painful parts.
The following perscription may be used:

℞ Phenacetin, - - - - gr.lxxv;
Lanolin, - - - - ʒvi;
Olive oil, a sufficient quantity.
To be rubbed about the inflamed part.
Or,
℞ Phenacetin, - - - - gr.lxxv;
Alcohol, - - - - Oii.
In this solution dip compresses and apply to the painful parts.

